# The impact of care of the elderly certificates of added competence on family physician practice: results from a pan-Canadian multiple case study

**DOI:** 10.1186/s12877-022-03523-4

**Published:** 2022-11-07

**Authors:** Rebecca H. Correia, Lawrence Grierson, Ilana Allice, Henry Yu-Hin Siu, Alison Baker, Janelle Panday, Meredith Vanstone

**Affiliations:** 1 Department of Health Research Methods, Evidence and Impact, Faculty of Health Sciences, McMaster University, Hamilton, Canada; 2 Department of Family Medicine, Faculty of Health Sciences, McMaster University, Hamilton, Canada; 3 McMaster Education Research, Innovation, and Theory (MERIT), Faculty of Health Sciences, McMaster University, Hamilton, Canada

**Keywords:** Geriatrics, Enhanced skill, Added competence, Credentials, Family medicine, Health policy

## Abstract

**Background:**

Family physicians serve an important role in the care of older adults, and have variable levels of training and comfort navigating this complex patient population. The Care of the Elderly (COE) Certificate of Added Competence offered by The College of Family Physicians of Canada recognizes family physicians with advanced expertise in older adult healthcare. We explored how COE training and certification impacts primary care delivery to older patients, including factors that impact group practice.

**Methods:**

We conducted a secondary analysis of multiple case study data to explore similarities and differences within and across cases. We defined cases as a practice or collective of family physicians working within a defined group of patients in an interconnected community. We analyzed semi-structured interview transcripts (*n* = 48) from six practice groups of family physicians across Canada using conventional (unconstrained, inductive) content analysis.

**Results:**

We identified similarities and differences in how COE family physicians function within their group practice and the broader healthcare system. In some cases, COE certifications increased patients’ access to geriatric resources by reducing travel and wait times. Some physicians observed minimal changes in their role or group practice after earning the COE designation, including continuing to largely function as a generalist. While family physicians tended to highly value their COE CAC, this designation was differentially recognized by others.

**Conclusions:**

Our findings highlight the impacts and limitations of COE training and certification, including an opportunity for COE family physicians to fill knowledge and practice gaps. As the number of older adults in Canada continues to grow and increasingly rely on primary care services, COE family physicians are uniquely positioned to strengthen the health system’s capacity to deliver specialized geriatric care.

## Background

Older adults seeking primary care services often present with greater care needs due to frailty, multimorbidity, functional decline, polypharmacy, and multiple care transitions [1–3]. Although family physicians provide the highest volume of medical services to older patients relative to all other specialty groups [4, 5], many report varying degrees of preparedness to and interest in providing geriatric-related care [4, 6, 7]. Caring for older adults is complex due to challenges at the individual, practice and system levels, largely related to care integration, collaboration, and coordination [8–12]. In countries with an aging population, there is a growing need to understand the nature of primary care provision for this group, including strategies to encourage high quality care and potential challenges to this goal [8, 13].

Barriers to delivering efficacious primary care to older adults may relate to the variable training and experience family physicians have with this population [6, 14–16]. In Canada, challenges in the care of older patients may reflect diversity of primary care practice scopes and organizations [11, 17–22], payment models [23, 24], practice locations and regions [25–27], and training and credentialing initiatives [28]. Given population-level demands to care for older adults and longstanding deficits in human health resources [29–31], there is a need to align medical education and primary health care systems to adequately and competently care for this patient population [5].

Recognizing a gap in formal training opportunities for family physicians to increase their knowledge and skills to care for medically complex older patients, the professional association and certifying body of family physicians in Canada (i.e., the College of Family Physicians of Canada, CFPC) established the Care of the Elderly (COE) training and certification program [32–34]. Since its inception, over 414 family physicians in Canada have earned the COE designation through residency training or practice experience and professional development [14, 35, 36]. Eighteen priority topics in medical care of the aged underpin the training and evaluation criteria for COE programs [14, 35, 37]. While COE programs may be one avenue to increase capacity and competence within the healthcare system to care for aging patients [13, 14, 31, 38–40], the impacts of COE programs on primary care delivery and group practice is largely unknown [41].

This study aims to examine the impact of the COE CAC on how groups of family physicians provide care to older patients through an in-depth analysis of multiple cases across Canada. This investigation is organized around the research question: How does COE training and certification impact the way primary care is delivered to older patients? By examining the roles of family physicians – including those with and without COE designations – in caring for older patients, this study will provide insights for continued investments in geriatric-focused medical education and primary care organization.

## Methods

### Study design

Previously, we conducted a multiple case study to examine the impacts of four Certificates of Added Competence (CAC) programs (i.e., COE, Family Practice Anesthesia, Palliative Care, and Sports and Exercise Medicine) on the provision of comprehensive care in Canada, explore how the CACs affected the experiences of members, and consider the potential benefits and risks of the programs [34, 42]. The current study is a secondary analysis of the six instrumental cases from the multiple case study, which centres specifically on the COE CAC and primary care delivery to older patients [34, 43]. Elsewhere we have published focused analyses on the Sport and Exercise Medicine and Emergency Medicine CACs [44, 45]. A multiple case study design facilitated our in-depth exploration of variation and diversity within and across cases, with consideration of the broader organizational context and related conditions that may influence its success [46, 47].

**Fig. 1:**
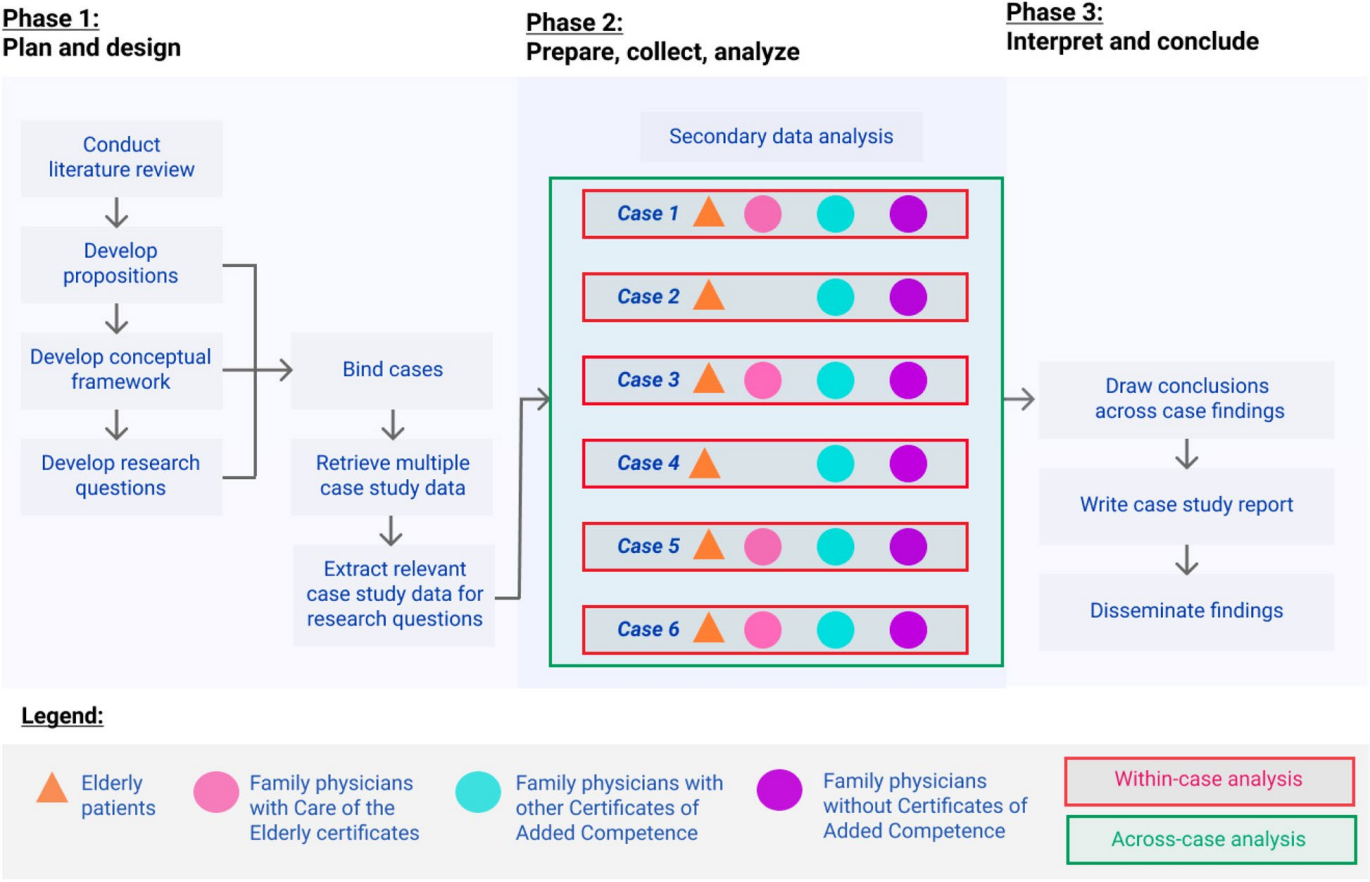
Overview of multiple case study design and approach. Adapted from. [46, 49, 50]

Cases were defined as a practice or collective of family physicians (including those with and without CACs) working within a defined group of patients in an interconnected community (Fig. 1). For each case, we examined the broader contextual factors impacting the COE program and primary care delivery, which diversified the breadth and depth of our inquiry [46, 48]. With the embedded nature of our study, physicians were positioned within the case and broader context to examine potentially contrasting aspects in how they care for older patients [46]. This study was bounded within Canada’s publicly funded health care system, which involves the delivery of medical care through multiple geographic, political, social, and economic contexts[11, 27].

Six instrumental cases were sampled using a variety of theoretically relevant features of family medicine practice (e.g., remuneration types, practice models, geographic locations, rurality, Francophone/Anglophone, and interprofessional mix) [34]. Older patients were part of the practice populations in all cases. A comprehensive description of recruitment/sampling strategies is described in the multiple case study from which this secondary study originates [34].

### Data collection

The data collection methods used in the original multiple case study are described elsewhere [34]. For both phases of this work, the primary data source included key informant interviews within each case. Semi-structured interview guides were developed for family physicians, residents/learners, and administrators/managers who worked within primary care settings [34]. The interview guides were organized around the theoretical propositions of the original multiple case study [46, 48]. For this secondary analysis, the propositions listed in Table 1were developed to bind the study, inform the research questions, guide the analysis, and ensure a thorough exploration of the phenomenon of interest [48]. Interviews were conducted by a research assistant (IA), recorded, and transcribed verbatim.

**Table 1:** Research questions and propositions

Research questions	Primary	How does COE training and certification impact the way primary care is delivered to older patients?
	Secondary	Within primary care settings, what is the context of geriatric care in Canada? What factors influence the ways in which groups of family physicians care for older Canadians?
Propositions	1. Across primary care groups, there are substantial practice differences among COE family physicians in how they care for older patients 2. Within and across primary care groups, the practice of COE family physicians substantially differs from other family physicians in how they care for older patients (e.g., functioning as a consultant, referral patterns, patient roster) 3. Within primary care groups, COE family physicians increase capacity to deliver geriatric care to older patients (e.g., expanded skillset, educational resource)	

### Data analysis

Before conducting the data analysis, we assessed the fit between the primary dataset from the original multiple case study and the research questions posed for our secondary analysis [43]. In our initial read of all interview transcripts, we found that care of older adults was integral to every case – suggesting the cases were widely relevant to the research objectives and propositions of our secondary analysis [43, 48].

We performed a descriptive content analysis using a staged coding process [48]. The first stage involved immersion, condensation, and summarization, allowing for subsequent iterations of analysis to develop categories and sub-categories [51]. While conducting the analysis, we frequently referenced the propositions to focus the analysis within the study’s scope and increase our confidence in the findings as the propositions and rival propositions were addressed, accepted, or rejected [46]. The second phase involved a thematic cross-case analysis to identify cross-cutting themes and explore the similarities and points of distinction across the six cases [52]. The analysis began in an unconstrained inductive fashion, given the lack of plausible existing theory, with findings from each case informing the approach to coding subsequent cases [53].

The primary investigator (RC) coded each interview transcript and documented analytic memos to capture potential avenues for analysis and patterns [46]. A second coder (JP) applied the codebook to a random sample of highly relevant transcripts. The second coder checked the interpretations against the data from a different perspective, resulting in iterative revisions to the coding structure. Emerging themes, patterns, and other findings were discussed among the full research team, which included a generalist family physician (AB), a COE family physician (HS), and health systems and policy researchers (RC, JP, MV, LG, and IA). The data were managed through NVivo version 12.

### Methodological quality and rigor

As per Yin, four aspects of methodological quality and rigour were maximized – construct validity, internal validity, external validity, and reliability [46]. We ensured construct validity through member checking, whereby two family physicians who provide care to elderly patients (HS and AB) reviewed the findings to ensure they resonated with their experiences [47, 48]. Pattern matching and explanation-building were used to establish internal validity throughout the analysis [46]. Multiple researchers coded the data derived from the interview transcripts and compared patterns that we observed with ones that we predicted in our protocol; similarities suggested greater internal validity [46]. Through explanation-building, we developed theoretical statements, compared our case findings to these statements, and revised the propositions accordingly. By engaging in this iterative process, our confidence in the findings is increased as the number of propositions and rival propositions were addressed and accepted or rejected. External validity was reinforced through cross-case synthesis and analytic generalization[46]. As per case study research, our goal was to expand and generalize theories. We drew comparisons across cases, which were re-contextualized and re-integrated into themes to explore theoretical relationships. Reliability was supported through data management and using a case study protocol [46]. Each of these case study strategies also helped support the trustworthiness of the research study by establishing credibility (through dual coding and peer debriefing), transferability (explanation-building, cross-case synthesis, and analytic generalization), and confirmability (reflexivity and documenting the chain of evidence).

### Data sufficiency

Due to the secondary nature of this study after case selection had occurred, the assessment of data sufficiency relates to a rich description of the selected cases [46]. The original multiple case study generated a highly detailed, contextualized, and substantial volume of data. We operationalized “completeness” by developing propositions within a case and testing them across cases, which achieved comprehensiveness in our understanding of the case [46]. Triangulation of findings occurred among interview participants within and across each case and the two coders who conducted the analysis [46, 48]. Member checking affirmed that the explanations offered by the data were comprehensive and sufficient to also encompass the experiences of those who provide care to elderly patients [47, 48]. We also maintained a chain of evidence and audit trail that helped support data sufficiency.

### Ethical considerations

The original multiple case study was reviewed and approved by the Hamilton Integrated Research Ethics Board (HiREB #5151). Each participant in this study provided verbal and written consent. We rationalized our secondary use of the data based on the alignment of the original and secondary research questions, and the similar focus across both phases of the research [43].

## Results

Forty-eight physicians, resident trainees, and administrators were interviewed across a variety of regions in Canada, including 39 family physicians, 6 resident trainees, 2 administrative staff, and 1 geriatrician (Table 2). All groups of family physicians cared for elderly patients, affirming the relevance of each case. Twenty family physicians were CAC holders, 6 held COE CACs, and 11 were enhanced skills family physicians. Except Case 2, each case included at least one COE holder. Full case descriptions are detailed in the original multiple case study [34].

**Table 2:** Overview of case and participant features

Case	Descriptive Context										Interview Participants							
	**Geography**				**Region**	**Training site**		**Tertiary-level hospital**	**Inpatient care**		**Number of participants**	**Family physician**				**Geriatrician**	**Resident trainee**	**Administrative Staff**
	Urban	Suburban	Rural	Remote		Community	Academic		Specialist-led	FP-led		COE holder	Other CAC	Generalist^*^	Enhanced skills			
1	X				Central		X	X	X		6	1	2	0	2	0	1	0
2			X		Prairies		X	X		X	15	0	5	2	5	0	1	2
3	X				Atlantic		X			X	8	2	1	3	0	0	2	0
4				X	Northern Territories		X			X	5	1	3	1	0	0	0	0
5			X		Pacific		X			X	8	1	1	1	3	1	1	0
6		X			Central	X		X	X		6	1	2	1	1	0	1	0
**Total**	**2**	**1**	**2**	**1**		**1**	**5**	**3**	**2**	**4**	**48**	**6**	**14**	**8**	**11**	**1**	**6**	**2**

*FP* Family physician, *CAC* Certificate of Added Competence, *COE* Care of the Elderly

^*^Does not hold a CAC

### Impacts of COE programs

We observed substantial differences in how individual COE family physicians function and deliver medical care collaboratively in different settings, contexts, and regions across Canada. While the program consistently enabled providers to increase their capacity to deliver geriatric-focused care, there were differences in the perceptions and attitudes towards COE designations. Common features within the cases included factors motivating physicians to pursue COE training. There were some variations in the roles of COE family physicians delivering care to older patients, remuneration, and opportunities for leadership, teaching, and scholarship.

We summarize the interconnected ways in which COE training and certification influences the care of older persons across the patient, provider, and practice levels.

## Patient-level

Our analysis identified four patient-level impacts of COE programs: access to geriatric care, continuity of care, comprehensive care, and community-adaptive care.

### Access to geriatric care

Access to COE family physicians was described as a means to reduce wait times for older patients and streamline referrals when additional care was warranted (Cases 2, 3, 5, 6). In turn, this lessened the burden on patients to travel and seek specialist care elsewhere, especially for those in rural or remote regions (Cases 1, 2, 3, 5), and “*decrease*[d] *the wait list for specialists because the specialists* [were] *able to take on all those really complex cases*” (Case 6, COE family physician).

COE programs enabled some family physicians to deliver specialized geriatric services in their primary care practices, like comprehensive geriatric assessments or memory clinic consultations (Case 1). Many COE family physicians perceived their role as building capacity within their group practice by functioning as a skilled resource during rounds and informal consultations (Cases 1, 2, 3, 5).

### Continuity of care

Most cases reflected on the longitudinal nature of the patient-family doctor dyad and discussed how these *“intimate” *relationships (Case 3, COE family physician) evolve as patients age (Cases 3, 4, 6). COE programs were praised for facilitating care continuity by enabling primary care teams to adequately support patients in the community as they age (Case 3).

For others, the episodic care provided by specialists and enhanced skills physicians was perceived as advantageous to preserve the patient-family doctor relationship when diagnosing stigmatized health conditions (e.g., dementia) or discussing lifestyle changes (e.g., driving) (Cases 3, 4). Across multiple cases there were mentions of relief at the opportunity to refer these difficult aspects of care to a less-connected physician, such as one physician who described being *“encouraged by the geriatricians to refer for that reason, just because family medicine is really about long-term relationships” *(Case 3, resident physician).

*“I have friends who are family physicians, they thank me for writing that letter. And they know I get beaten up for it, but the thing is, I’ve seen emotional reactions. […] These people are yelling at me, they’re threatening me, and all that. But here’s the thing. I’m not the most important physician for these people. They can hate me, but […] the most important person for a patient, really, is the community family physician. […] So, I don’t like it, but I would do it because I feel that it is for the greater good in terms of patient care. So, they get to keep that relationship.” – Case 3, COE family physician.*

### Comprehensive care

COE programs impacted the delivery of comprehensive care for older patients, both while physicians pursued the training and after they earned the designation. In communities with fewer specialist resources, COE family physicians seemed to transition away from their generalist family medicine practice to care for older patients more intensively (Cases 3, 5). Participating COE family physicians in more urban areas appeared to sustain their role and general family medicine practice after earning the designation (Cases 1, 6). Others suggested that the COE family physician’s age, rather than their location, influenced their pursuit of a focused practice: “*If you think of comprehensive care […], I would say that my generation it’s a given, that’s what we do. The younger generation I would say it’s not so much a given. There are GPs who just do obstetrics and then they don’t want to see you when you grow old*” (Case 4, COE family physician). One family physician shared how their core family medicine training and former role delivering comprehensive care influences their current focused practice in COE:

*“I think it makes me feel a little bit more responsible for the patients than maybe I should be, because I tend to do follow-ups a little bit more than probably I should. I have to constantly remind myself that I should give these people back to the family doctor. I have this big pile of new consults and if I keep following the same people over and over again, I’m not going to be able to do that. So, it colours what I do…” – Case 2, COE family physician.*

### Community-adaptive care

Across cases, COE credentials enabled providers to practice in more diverse health care settings. COE family physicians described their practice in outpatient clinics (Cases 1, 3), inpatient units (Cases 1, 3), memory clinics (Cases 1, 3), home care (Cases 1, 3, 4, 6), and long-term care (Cases 1, 2, 3, 4, 5, 6).

## Provider-level

Provider-level factors speak to how COE programs impact family physicians’ competence and feelings of validation, identity and recognition, multidisciplinary collaborations, pursuit of scholarship and leadership, hiring choices, and work-life balance.

### Competence and validation

After earning the designation, many COE family physicians expressed greater confidence to care for older patients (Cases 1, 2, 5, 6) and felt less like an “*imposter*” (Case 2, COE family physician). The COE designation provided family physicians with a formal title that empowered them to conduct complex assessments, lead advocacy initiatives, pursue research grants, and converse confidently with specialists and administrators (Cases 1, 3, 4, 5, 6). Some physicians believed the COE CAC “*validated the work that* [they] *had done and it actually gave* [them] *the credentials*,” and allowed for “*other people* [to] *recognize that* [they] *knew what* [they were] *doing*” (Case 2, COE family physician). Among those who maintained a general family medicine practice, COE training was regarded as advantageous to care for patients across the age spectrum because the physicians could proactively “*have discussions* [about] *things that* [they] *see are issues as people get older*” (Case 1, COE family physician).

### Identity and recognition

There were substantial differences in how COE family physicians perceived their role within the health care system, and how others regarded or utilized their expertise. Some colleagues who worked alongside COE family physicians revealed they did not understand or appreciate what the CAC represented (Cases 2, 3, 6). A generalist colleague shared, “*I think they’re still considered family physicians*” when asked about their COE colleagues (Case 3, Family physician), whereas a COE holder expressed, “*I’m really not a family physician, as well, I do a lot of internal medicine*” (Case 3, COE family physician). Intrinsically, some COE family physicians believed their CAC was not recognized by colleagues and that the designation did not impact their professional roles or opportunities (Cases 3, 4). A few COE family physicians spoke about the importance of maintaining some aspect of their family medicine practice to maintain their core skills as generalists because they identify as a family physician “*first*” and a COE holder “*second*” (Case 1, COE family physician).

### Multidisciplinary collaborations

There were rich discussions in most cases about how health care professionals from multiple disciplines engage in the shared care of older adults, including those with family medicine, internal medicine, psychiatry, nursing, social work, recreation therapy, occupational therapy, and physiotherapy backgrounds (Cases 1, 2, 3, 5, 6). Collaborations amongst COE family physicians and geriatricians, in particular, afforded opportunities to “*talk about difficult cases and* [learn] *what* [they’re] *doing with them*,” (Case 3, COE family physician) and handover patients based on a physician’s area of interest or expertise. General family physicians applauded COE holders for “*extending* [their] *knowledge*” and giving “*that opportunity to manage up to where* [they] *feel, okay, now we need help and then* [they] *refer*” (Case 3, Family physician). In under-resourced areas, such as those lacking access to geriatric psychiatrists, COE family physicians were sought to assist with behavioural issues or dementia cases among older patients (Case 2). One participant envisioned COE family physicians functioning as an intermediary between generalists and specialists as “*that sort of mid-level access where it’s basically the first line of what it’s referred and a conversation of how then to escalate*” (Case 6, COE family physician).

### Scholarship and leadership

Family physicians in some cases described quality improvement (Cases 1, 5) or research activities (Case 5) they pursued having earned the COE designation, which were sometimes tied with academic appointments (Case 1). Participants in Cases 1, 5, and 6 spoke about clinical leadership opportunities that were afforded by the COE designation. For example, two COE family physicians described how they developed clinical management tools (Case 5) and rebuilt the geriatric program in a family health team (Case 6). One provider emphasized the importance of leveraging the COE designation for leadership, policy, and advocacy opportunities:

“*What you can [do] with a CAC is maybe you can talk to the Ontario Long Term Care Association and say, like ‘look I have this expertise, like I’m interested in doing some advocacy work.’ Or you can partner with a resident or a patient council, or when you apply for a grant it gives you that much more kind of like optics that you are engaged in this. So, I think that’s how I see my CAC is that I’m leveraging it for those things and not necessarily to get a more defined care of the elderly practice.*” – Case 1, COE family physician.

### Hiring choices

Participants in all cases reflected on the impacts of COE certifications on hiring choices and job security, although the opinions were mixed about whether the certificate was advantageous or necessary. Some believed the COE designation afforded credibility if they moved to a different practice location (Case 3), offered incentives like financial advantages (Case 3), or provided a competitive edge in the job market (Case 1). Despite population aging, one COE family physician recalled, “*some positions are quite competitive now in terms of job opportunities*. […] *It’s actually quite difficult to get a nursing home position*” (Case 1). One participant pursued the designation to maintain a competitive edge: “*So, I said for job security, if I want to work another 10 years, I better do it because these young guys are coming along*” (Case 4, COE family physician).

Additionally, for those who completed medical training outside of Canada, the COE credentialling process provided an opportunity to recognize intensive training in geriatrics obtained elsewhere (Cases 3, 6). One participant described how their training in geriatrics in the United States was not recognized by the regulatory body of physicians in Canada, which impacted the scope of their practice despite having adequate knowledge and skills: “*Because of the fact that I trained in the States they don’t recognize that here*. […] *I wasn’t able to take consults or anything like that though I had colleagues who did ask me to do so*” (Case 6, COE family physician).

### Work-life balance

Most cases described how the COE designation impacted the boundaries between their professional and personal lives. COE family physicians in Cases 3, 4, and 6 discussed the challenges of working excessive hours and balancing the competing demands of multiple roles and various practice settings. One newly qualified COE family physician credits the program for affording elasticity in their routine and medical practice organization: “*I’m not convinced that having a large family practice at this point is for me, especially because I have a young family at this time. So, I really enjoy the flexibility, for example, of nursing home work and the hours that can be flexible based on my family’s schedule*” (Case 1, COE family physician).

## Practice-level

Lastly, the COE certificate poses implications on the practice-level in terms of human health resources planning, functioning as a generalist or specialist, and remuneration.

### Human health resources planning

Physicians in all cases reflected on the need for specialized geriatric resources to meet growing, population-level demands. As articulated by one participant, the need for additional geriatric resources extends across the health system: *“The internal medicine geriatricians will not be able to keep up with geriatrics in Canada. The family doctors don’t have time to do geriatrics in their practice. And, I think that having family doctors with extra training in geriatrics or care of the elderly is going to be really necessary”* (Case 3, COE family physician). Some family physicians were motivated to pursue the COE designation to adequately care for the aging patients in their group practice (Case 4), while others intended to establish focused practices to counter daunting wait times inhibiting timely access to geriatric specialists (Case 3).

Interestingly, in regions where health care services were broadly under-resourced, the need for geriatric-focused providers was seldom discussed (Cases 2, 3). In these areas, other resources – like general family medicine – were paramount to address the diverse needs of the community.

### Continuum of generalist to specialist

COE family physicians inconsistently classified their expertise in caring for older patients along the continuum of ‘generalist’ to ‘specialist’. While some COE family physicians perceived their role as acting like an expert resource and have taken on specialized roles afforded by the CAC, others expressed disinterest in pursuing the COE CAC based on this premise:

*“I don’t know if I would need or, honestly, want the extra one because I think if I did do the Care of the Elderly, then there would probably be a reasonable expectation that I was going to provide extra services to the region. […] I've never really seen myself as a consultant, I've just seen myself as a general practitioner and my focus has always been my practice.” – Case 1, General family physician.*

COE holders expressed diverse interests to take on leadership roles in geriatric-focused care settings (e.g., nursing home director) (Case 3), undergraduate or postgraduate educational positions related to geriatrics (e.g., education program director) (Case 1), or establish a focused practice caring for older patients (Case 1). The flexibility to apply the COE designation in different professional pursuits affords ample opportunities for providers to follow special interests and advance their own careers.

### Remuneration

All cases included rich discussions of the financial implications of pursuing COE training and practicing after earning the designation. Many COE holders remarked on the costly process to step away from their family practice to pursue CAC training with little (Case 5) or no (Cases 1, 2, 3, 4, 6) financial incentives. Participants in Case 2 attributed the absence of COE family physicians in their jurisdiction to the lack of financial support incentivizing COE training or encouraging physicians to established focused practices for older adults.

The fee structures in which COE family physicians were compensated varied widely. All cases recognized the importance of salaried funding models to allow for extensive consultations and meetings with patients beyond the traditional “*15-min primary care appointment*” (Case 3, COE family physician). Some participants questioned differences in remuneration between COE family physicians and geriatricians (Cases 3, 5, 6), “*despite doing the same work*,” in areas where providers practiced collaboratively in similar settings (Case 3, COE family physician).

## Discussion

Our analysis of COE training and certification, based on the experiences of physicians and administrative staff across multiple cases in Canada, is one of the first efforts to explore the impacts of this program. Prior work examining the practice of COE family physicians has been largely descriptive [31, 31, 37, 38] or examined the impacts of COE programs broadly [34]. Ours is the first qualitative study we can identify examining the implications of COE training and certification on the primary care of older adults. A multiple case study design allowed for the identification and exploration of substantial differences in the practice of COE family physicians, as well as perceptions and attitudes towards the designation (both intrinsic and extrinsic). Despite differences in formal roles, clinical responsibilities, collaborations with other health care providers, and remuneration patterns, the COE designation was consistently regarded for enabling family physicians to increase the health system’s capacity to adequately care for older adults.

We identified factors at the patient, provider, and practice levels that explained how groups of family physicians collaborate in the shared care for older adults across different settings, contexts, and regions. We drew parallels between these factors and the Institute of Medicine’s “Ten C’s of Primary Care,” which has informed many competency and evaluation tools related to family medicine training, and the CFPC’s “Triple C Competency-based Curriculum,” which outlines teaching and assessment expectations for family physicians [54, 55]. Similarities between the impacts of COE programs and “Ten C’s of Primary Care” include calls for family physicians to demonstrate continuous, comprehensive, and coordinated care, participate in continuing education, ensure cost-effectiveness in decision-making, and collaborate with patients and other health care workers [54]. Similarly, the CFPC outlined goals for family physicians to exhibit competence in providing comprehensive care, adapting to community needs, and referencing the best available evidence – all of which were identified as patient- and provider-level impacts of COE training and certification [55]. Congruence across the impacts of COE programs and competency framing documents suggests that COE family physicians are employing their training and functioning in ways that align with the original intentions of their governing body.

The impacts of COE training and certification that we identified in this work align with the findings of the original multiple case study which examined four CAC domains. Similarly, the COE CAC and others were responsive in addressing needs within the community to increase access to under-resourced services, reducing the need to seek specialist care in other communities, and maintaining continuity between primary and tertiary care services[34]. The “risks” identified in the original multiple case study were echoed in this work – the COE program may decrease the number of family physicians willing to provide comprehensive care to patients, reduced morale towards generalist family medicine, and encourage credential creep whereby standard family medicine designations are devalued [34]. Our examination of COE family physicians, in particular, was distinct from the original multiple case study, in that the work of family physicians without formal enhanced skills training in geriatrics was not discounted.

Despite the widely recognized need for more geriatric-focused providers in Canada [31, 38], and the opportunity for COE family physicians to address this void [8, 56], our work validates challenges of differentiating between the care provided by geriatric generalists and specialists [49, 50, 57]. Caring for elderly patients is an important core competence for all family physicians. CAC holders are family physicians with a unique combination of generalist and enhanced skills, some of which may overlap with other geriatric clinicians [42]. Those who wish to increase the availability of skilled clinicians in this area should understand the ways in which family physicians without enhanced or specialized credentials and those with formal geriatrics training (i.e., COE family physicians, geriatricians, and geriatric psychiatrists) deliver complementary care to a shared patient population. For example, there is an opportunity for training and certification programs to specify the degree of geriatric-care skills for COE family physicians to acquire – relative to other geriatric-focused physicians – to differentiate their degree of expertise in caring for a shared patient population [58]. The original multiple case study identified four organizational models that further illustrates the collaborative relationships of general family physicians, those with enhanced skills or certificates, and specialists [34].

### Limitations

This secondary analysis was limited by the research questions, objectives, and scope proposed in the multiple case study in which the interview data originates [43]. Since activities like the sampling strategy, participant recruitment, constructing the interview guide, and conducting interviews occurred prior to this secondary study, we were limited to the breadth and degree of detail specified in the original interview transcripts to achieve our research objectives.

## Conclusions

This study identified and explored the impacts of COE training and certification on primary care delivery to older patients across multiple cases in Canada. This examination of COE family physicians’ contributions – across primary care groups and health care settings – demonstrated their diverse and invaluable roles increasing capacity for elder care and filling knowledge and practice gaps. As more family physicians become certified in COE, the ways in which primary care is delivered to older patients may change to reflect differences in collaborative care models, specialist referral patterns, and scopes of comprehensive care.

## Data Availability

The qualitative data analyzed during the current study are not available for use by other researchers due to ethical regulations restricting its use.

## Abbreviations

COE: Care of the Elderly
CFPC: College of Family Physicians of Canada
CACs: Certificates of Added Competence
